# Dihydrotanshinone I inhibits ovarian cancer cell proliferation and migration by transcriptional repression of PIK3CA gene

**DOI:** 10.1111/jcmm.15660

**Published:** 2020-08-29

**Authors:** Xiaoqing Wang, Xiao Xu, Guoqiang Jiang, Cuili Zhang, Likun Liu, Jian Kang, Jing Wang, Lawrence Owusu, Liye Zhou, Lin Zhang, Weiling Li

**Affiliations:** ^1^ Department of Biotechnology Basic Medical School Dalian Medical University Dalian China; ^2^ Department of Medical Oncology Dana‐Farber Cancer Institute and Harvard Medical School Boston MA USA; ^3^ Academy of Integrative Medicine Dalian Medical University Dalian China

**Keywords:** dihydrotanshinone I, migration, ovarian cancer, PIK3CA, proliferation

## Abstract

Dihydrotanshinone I (DHTS), extracted from Salvia miltiorrhiza, was found to be the most effective compound of tanshen extracts against cancer cells in our previous studies. However, the therapeutic benefits and underlying mechanisms of DHTS on ovarian cancer remain uncertain. In this study, we demonstrated the cytocidal effects of DHTS on chemosensitive ovarian cancer cells with or without platinum‐based chemotherapy. DHTS was able to inhibit proliferation and migration of ovarian cancer cells in vitro and in vivo through modulation of the PI3K/AKT signalling pathways. Combinatorial treatment of DHTS and cisplatin exhibited enhanced DNA damage in ovarian cancer cells. Overall, these findings suggest that DHTS induces ovarian cancer cells death via induction of DNA damage and inhibits ovarian cancer cell proliferation and migration.

## 1 INTRODUCTION

Ovarian cancer (OVCA) accounts for 225 000 new cases and 140 200 cancer‐specific deaths each year globally.^1^, ^2^ As the leading cause of death from gynaecological malignancies, ovarian cancer has attracted considerable awareness in the Western world, although the incidence and survival rates of ovarian cancer vary by country.^3^, ^4^ It is estimated that 75% of ovarian cancer patients present with evidence of metastatic spread beyond the ovaries at the time of diagnosis and require combined debulking surgery and chemotherapy.^5^ Ovarian cancer is a chemosensitive disease; about 75% of patients with advanced ovarian cancer (International Federation of Gynaecological Oncology [FIGO] stage III–IV) respond to front‐line paclitaxel‐platinum treatment.^5^ Platinum‐based chemotherapy improves both progression‐free and overall survival in all patient subgroups.^6^, ^7^, ^8^, ^9^ However, most of these patients will eventually relapse with a median progression‐free survival of 18 months^9^ and survival at 5 years is currently less than 30% (http://www.cancer.org).

Limitations of platinum‐based chemotherapy, such as drug resistance and non‐specific cytotoxicity, impel cancer biologists to identify more specific therapeutic agents for patients with ovarian cancer. Given that 49% of all internationally approved anti‐cancer drugs from the 1940s to 2014 were either natural products or direct derivatives,^10^ identification of novel anti‐cancer compounds from medicinal herbs has progressively gathered momentum over the past decades.^11^, ^12^ Tanshinones, first identified in the 1930s, are a class of lipophilic abietane diterpene compounds extracted from the dried root of Salvia miltiorrhiza. Tanshinone IIA, cryptotanshinone, tanshinone I and dihydrotanshinone I (DHTS) are four major constituents of tanshinones.^13^ Recently, several studies have shown that tanshinone I, dihydrotanshinone I and tanshinone IIA could exert pro‐apoptotic and cytotoxic effects on a number of human cancer cell lines^14^, ^15^ and also inhibit epithelial‐mesenchymal transition (EMT) and migration.^16^, ^17^ Induction of apoptosis is well‐accepted as one of the most promising therapeutic strategies for cancer treatment.^18^, ^19^ Previous studies revealed the pro‐apoptotic ability of DHTS in human erythroleukemia, glioma, osteosarcoma and colorectal cancer in both in vitro and in vivo settings.^20^, ^21^, ^22^, ^23^, ^24^, ^25^ In addition, emerging evidence suggests that DHTS treatment attenuated cell migration by down‐regulating adhesion molecules VCAM‐1 and ICAM‐1 in osteosarcoma cells.^22^ Research on DHTS has revealed its beneficial effects with regard to apoptosis induction and migration suppression. However, the therapeutic benefits and underlying mechanisms of DHTS on ovarian cancer remain uncertain.

In this study, we evaluated the therapeutic efficacy of DHTS in the treatment of ovarian cancer. Our findings demonstrated the anti‐tumour effects of DHTS against chemosensitive ovarian cancer cells (A2780, OV2008) with or without platinum‐based chemotherapy. This compound exhibited minimal cytotoxicity towards immortalized normal ovarian surface epithelial cells (IOSE80) but remarkably inhibited the proliferation of abnormal ovarian cancer cells. Cell death pathway analysis revealed an impaired expression of PIK3CA (encoding PI3K catalytic subunit p110α) gene. We then demonstrated that DHTS inhibited proliferation and migration of OVCA cells through modulation of PI3K/AKT signalling pathways. Furthermore, combinatorial treatment of DHTS and cisplatin promoted DNA damage in OVCA cells. Our study demonstrated DHTS as a novel therapeutic agent of ovarian cancer, which disrupts PI3K pathway, and sensitizes cancer cells to platinum by inducing more DNA double‐strand breaks (DSBs).

## 2 MATERIALS AND METHODS

### 2.1 Reagents and antibodies

Dihydrotanshinone I (DHTS > 98%) powder (D0947) was purchased from Sigma‐Aldrich (St. Louis, MO, USA), and a stock solution of DHTS at 100 mM was prepared in DMSO (Sigma) and stored at −20°C. Cisplatin was purchased from Sigma‐Aldrich. The final concentration of DMSO was 0.1% in all treatment groups and had no effect on cell viability. The chemical formula of DHTS is C18H14O3. Dulbecco’ s modified Eagle’ s medium (DMEM), Foetal Bovine Serum (FBS) and phosphate‐buffered saline (PBS) were obtained from Gibco Thermo Fisher Scientific (New York, NY, USA). The primary antibodies against AKT, P‐AKT, PIK3CA and β‐actin were obtained from Protein tech Group Inc (Chicago, IL, USA). The enhanced chemiluminescence (ECL) kit was from Amersham Life Science, Inc (New York, NY, USA). Matrigel and transwells were from BD Biosciences (San Jose, CA, USA). Thiazolyl blue tetrazolium bromide (MTT) was obtained from Sigma Chemical Co. (St. Louis, MO, USA).

### 2.2 Cell culture

Human ovarian cancer cell lines, A2780 and OV2008, were purchased from American Type Culture Collection (ATCC). Normal ovarian epithelial cell line IOSE80 was obtained from the Canadian Ovarian Tissue Bank. Cells were maintained in DMEM medium supplemented with 10% FBS and 1% penicillin/streptomycin (streptomycin 100 μg/mL, penicillin 100 U/mL) at 37°C with 5% CO2 in a humidified incubator.

### 2.3 Cell viability assay

The effect of DHTS on the viability of cells was detected by MTT assay. Cells were seeded into 96‐well plates (~1 × 10^4^/well) and treated with various concentrations of DHTS (0,1, 2, 4, 6, 8 and 10 μM). At the indicated time‐points, the old medium was replaced with 100 μL of fresh medium containing 20 μL of MTT solution (5 mg/mL in PBS). After 4 hours of incubation at 37°C, formazan crystals were solubilized with 150 μL DMSO in each well. The absorbance levels for each sample at 540 nm were measured using a Multiskan Ascent plate reader (Thermo Electron, New York, NY, USA). The data were duplicated five times at least.

### 2.4 Wound healing assay

Wound healing assay was performed to evaluate the migration of ovarian cancer cells. Briefly, cells were seeded into 24‐well plates and grown until confluent state and then were scratched by using P1000 sterile tips. The plates were rinsed twice with sterile PBS to remove detached cancers cells. Fresh complete cell culture medium was added with various concentrations of DHTS. Cell migration was observed under a phase‐contrast microscope at 100 × magnification at 0 and 24 h post‐scratch. Migrated cells in the denuded area in each of six random fields were measured and quantified with a computer‐assisted microscope.

### 2.5 Transwell migration and invasion assay

Cell migration and invasion were evaluated by the transwell assay. For the transwell migration assay, ovarian cancer cells treated with DHTS were seeded into the upper chambers with FBS‐free medium at a density of 5 × 10^4^ cells per well and 600 μL of complete growth medium with 10% FBS was placed in the lower chamber as a chemoattractant. For the transwell invasion assay, 1 × 10^5^ cells per chamber were plated. Cells were allowed to invade through the Matrigel‐coated inserts. After 24 hours incubation at 37°C, noninvading cells remaining on the upper side of the chamber were removed with cotton swabs. Cancer cells that on the underside of the filter were stained with 0.1% crystal violet (Sigma) for 10 minutes and counted under a microscope at 200 × magnification. Six random fields of each transwell membrane were counted and averaged.

### 2.6 Microarray expression analysis

Ovarian cancer cells were treated with either DHTS or vehicle for 24 h and harvested for total RNA extraction by using TrizolTM reagent (Life Sciences). The RNA concentration and quality were evaluated by NanoDrop spectrophotometer (Thermo) and denaturing agarose gel(1.5%) electrophoresis. Cancer cell cDNA was synthesized through RT performed with an RT2 First Strand Kit (Qiagen) according to the manufacturer's instruction. Cell cDNA was created using an Affymetrix WT PLUS Reagent Kit. Array hybridization and washing was performed using GeneChip® Hybridization, Wash and Stain Kit (Cat#900720, Affymetrix, Santa Clara, CA, US) by following the manufacturer's instructions. Arrays were scanned using Affymetrix GeneChip® Scanner 3000 (Cat#00‐00213, Affymetrix, Santa Clara, CA, USA). Command Console Software (Affymetrix) was used to control the scanner and summarize probe cell intensity data (CEL file generation). Then raw data were normalized by Expression Console.

### 2.7 Colony formation assay

Cells were seeded in six‐well plates at a density of 500 cells per well. Then, medium with DHTS or vehicle was changed every 3 days for ~ 15 days when most of the colony contained more than 50 cells. After cold PBS washing, the colonies were fixed using 70% ethanol and stained with 0.1% crystal violet dissolved in 10% ethanol at room temperature. Then, the counts of cell colonies were manually scored, and the images were recorded under a computer‐assisted microscope.

### 2.8 Real‐time quantitative polymerase chain reaction (PCR) analysis

Real‐time PCR was used to quantify the cDNAs of interest using Taqman® Gene Expression Assay and Applied Biosystems 7900HT Fast Real‐Time PCR System. PCR reactions were prepared in a 20 μL volume containing 1 μL specific 20X Taqman Gene Expression Assay, 10 μL 20X Master Mix, 4 μL cDNA template (~100 ng) and 5 μL RNase‐free water. Routine real‐time PCR conditions were as followed: AmpliTaq Gold Enzyme activation at 95°C for 10 min, then 40 cycles of DNA denaturation at 95°C for 10 minutes, primer annealing and DNA polymerase extension at 60°C for 60 seconds. The primer sequences for the PIK3CA transcript were 5'‐TCCAGTCACTGTGCTGCTTC‐3' and 5'‐GCAAGGGAAAAGGGAGTCTT‐3'. The primer sequences for the DDB2 transcript were 5'‐TCAAGGACAAACCCCACCTTC‐3' and 5'‐GTGACCACCATTCGGCTACT‐3'. The primer sequences for the HGF transcript were 5'‐GGGGACGATACTGTCCTGAA‐3' and 5'‐GTCCCTCAGTGCACATCTCA‐3'. Each experiment was repeated at least thrice.

### 2.9 Western blot

Total protein was isolated from the cells using RIPA lysis buffer (Beyotime, China) with protease inhibitor cocktail and PMSF (Biocolors, China) at a proportion of 1:100. Equal amounts of protein were loaded on a 10% SDS‐PAGE gel. The lysates were resolved by electrophoresis (120 V for 1.5 hours) and transferred onto polyvinylidene difluoride (PVDF) membranes (Bio‐Rad, Hercules,California, USA). Membranes were blocked in 5% non‐fat milk for 1 h at room temperature and then incubated with different primary antibody at appropriate dilution in blocking buffer overnight in cold room. This was followed by incubation with relevant secondary antibodies for 1 hour at room temperature. Protein bands were visualized using the Chemiluminescent ECL assay kit (Amersham Life Sciences, Buckinghamshire, UK) for each group in a Bio‐Rad ChemiDoc XRS + image analyser. Protein expression levels were quantitatively determined by using ImageJ software (National Institutes of Health, Bethesda, MD, USA). β‐actin was used as internal reference for protein expression.

### 2.10 Zebrafish cancer model

Fertilized zebrafish eggs were incubated at 28°C in Danieau's solution and were raised under standard laboratory conditions. Twenty‐four hours post‐fertilization fish embryos were incubated with water containing 0.2 mM 1‐phenyl‐2‐thiourea (Sigma) to prevent pigmentation. At 48 hours post‐fertilization, zebrafish embryos were dechorionated with help of a sharp tip forceps and anaesthetized with 0.04 mg/mL of tricaine (MS‐222, Sigma). Anaesthetized embryos were transferred onto a modified agarose gel for microinjection. A2780 cancer cells were cultured at 37°C to 80% confluency, detached using Trypsin‐EDTA solution (Sigma), washed with DPBS (Gibco, Invitrogen) and further incubated for 20 min at 37°C in serum‐free culture medium with 0.05% DiD (Vibrant, Invitrogen). After an incubation of 20 min, cells were washed twice and further incubated for 5 h in media containing 0.25 μM bosutinib. Cells were washed twice and re‐suspended in DPBS for injection. Using Nanoject II (Drummond) injector, 200 cells were injected into the yolk of each embryo. After injection, the fish embryos were immediately transferred into housing‐keeping water. Injected embryos were kept at 28°C and were examined every other day for monitoring tumour growth and invasion using a fluorescent microscope.

### 2.11 Statistical analysis

Data are presented as means ± standard error of the mean (SEM) for three independent experiments. Statistical differences between different groups were analysed by using Student's t test by GraphPad Prism 5.0 (San Diego, CA, USA). A significant difference was considered at *P* < .05.

## 3 RESULTS

### 3.1 DHTS exhibits cytotoxicity to human ovarian cancer cells but not normal ovarian epithelial cells

**FIGURE 1:**
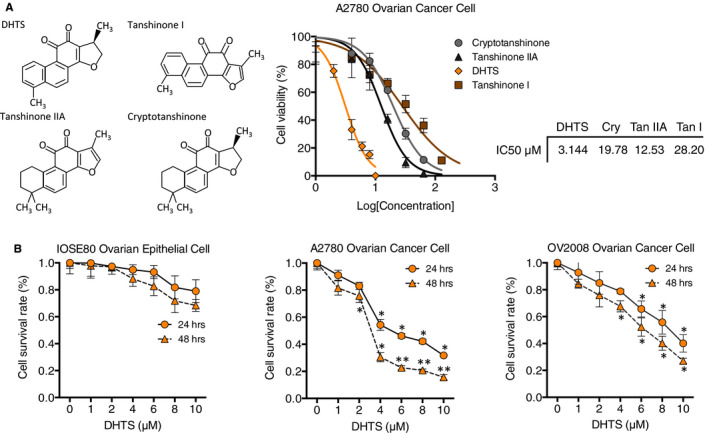
Dihydrotanshinone I selectively attenuates proliferation of SOC cells but not normal ovarian epithelial cells. A, IC50 of four compounds of the tanshinone family in human ovarian cancer cell line A2780. Ovarian cancer cells were treated with various concentrations of DHTS, tanshinone IIA, tanshinone I and cryptotanshinone for 48 h. Then, cell viability was determined by MTT assay. B, Anti‐proliferative effect of DHTS in IOSE80, A2780 and OV2008 cells by MTT assay. Cell lines were treated with various concentrations of DHTS (0, 1, 2, 4, 6, 8 and 10 μM) for 24 h or 48 h. Data represent means ± SEM of triplicate samples. *P < .05 and **P < .01 vs. untreated control

Cytotoxicity of four major constituents of tanshinones (DHTS, tanshinone IIA, tanshinone I and cryptotanshinone) was evaluated by MTT viability assay in A2780 human ovarian cancer cell line, which is a standard ovarian carcinoma (SOC) line and has been extensively used in research on ovarian carcinogenesis.^26^, ^27^ Among the four herbal compounds, we observed that DHTS exhibited the most cytotoxic potency on A2780 ovarian cancer cells with IC50 below 10 μM at 48h post‐treatment (Figure 1A), while cryptotanshinone, tanshinone IIA and tanshinone I were less potent. According to the in vitro activity of DHTS, we used DHTS to further exploit this bioactivity on the viability of another SOC cell line and normal ovarian epithelial cells. As shown in Figure 1B, ovarian cancer cell lines OV2008 and A2780, as well as normal ovarian cells IOSE80 were treated with different concentrations of DHTS (0, 1, 2, 4, 6, 8 and 10 μM) for 24 hours or 48 hours. DHTS did not induce significant cell death of normal ovarian epithelial cells IOSE80 (*P*> .05), while DHTS remarkably killed A2780 and OV2008 ovarian cancer cells in a dose‐ and time‐dependent manner (*P* < .05). The IC50 values of DHTS were 5.32 ± 0.42 and 3.14 ± 0.23 μM for A2780 cells, and 8.32 ± 0.54 and 5.21 ± 0.33 μM for OV2008 cells at 24 hours and 48 hours post‐treatment, respectively. All together, these data indicate that the DHTS specifically reduces cell viability of ovarian cancer cells but not non‐malignant ovarian epithelial cells.

### 3.2 DHTS inhibits migration and invasion of ovarian cancer cells

**FIGURE 2:**
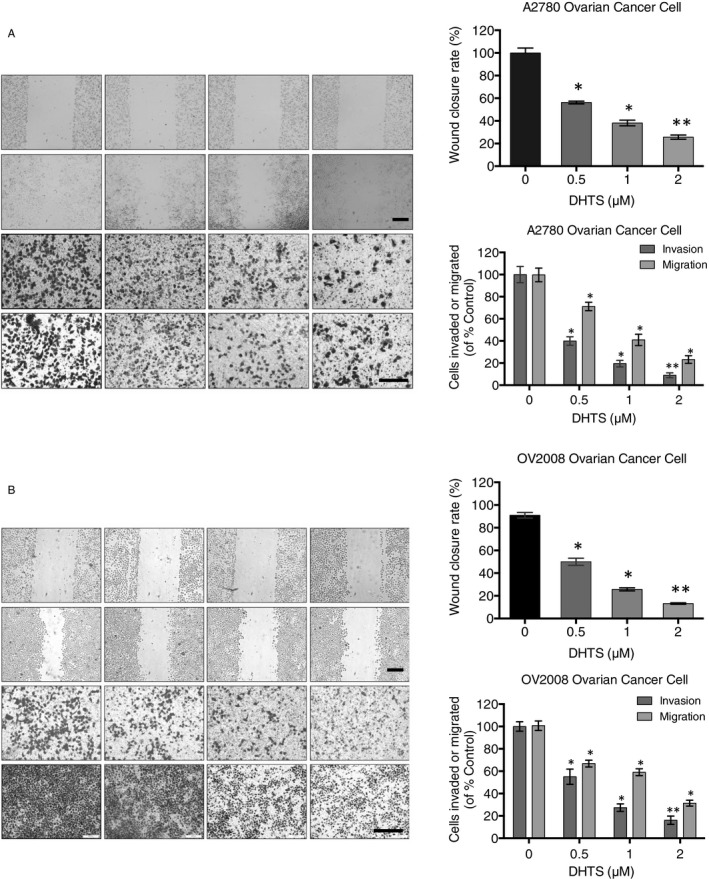
DHTS suppresses migration and invasion in A2780 and OV2008 ovarian cancer cells. (A, B) Effect of DHTS treatment on migration and invasion in A2780 and OV2008 ovarian cancer cells. Cancer cells were treated with different concentrations of DHTS (0, 0.5, 1 and 2 μM) for 24 h, followed by wound‐healing assay (top) and transwell chamber migration and invasion assays (bottom). Bar scale = 200 μm, *P < .05 and **P < .01 vs. untreated control

Women with malignant or even low malignant ovarian cancer are at a very high risk of metastasis, which involves cell migration and invasion during the process and can be fatal to patients.^22^, ^28^ A2780 and OV2008 cancer cells, with loss of PTEN and PIK3CA E545K mutation,^29^, ^30^, ^31^ are highly malignant with strong migration and invasion abilities. To evaluate whether DHTS exhibits anti‐migration and anti‐invasion effects, scratch wound healing and transwell chamber assays were performed at different low concentrations (0.5 μM, 1 μM, 2 μM) of DHTS. As shown in Figure 2A and B (upper panel), the wound closure rates of A2780 and OV2008 cancer cells decreased by 40%‐80% in response to DHTS treatment (0.5‐2 μM), comparing with control (DMSO) (Figure 2A, B, right panel, *P* < .01). Consistent with the wound healing assay, DHTS also significantly inhibited ovarian cancer cell migration and invasion, as determined by transwell chamber assays, in a dose‐dependent manner (Figure 2A, B, lower panel). Treatment with 0.5‐2 μM DHTS inhibited migration by 39%‐68% in A2780 cells, and 25%‐61% in OV2008 cells, as compared to controls, respectively. Similarly, cancer cell invasion was suppressed by 23%‐81% in A2780 and 41%‐72% in OV2008 (Figure 2A, B, right panel). These results suggested that DHTS can suppress the migratory and invasive abilities of ovarian cancer cells in a concentration‐dependent manner.

### 3.3 Gene expression profiling identified PIK3CA as target gene of DHTS in ovarian cancer cells

**FIGURE 3:**
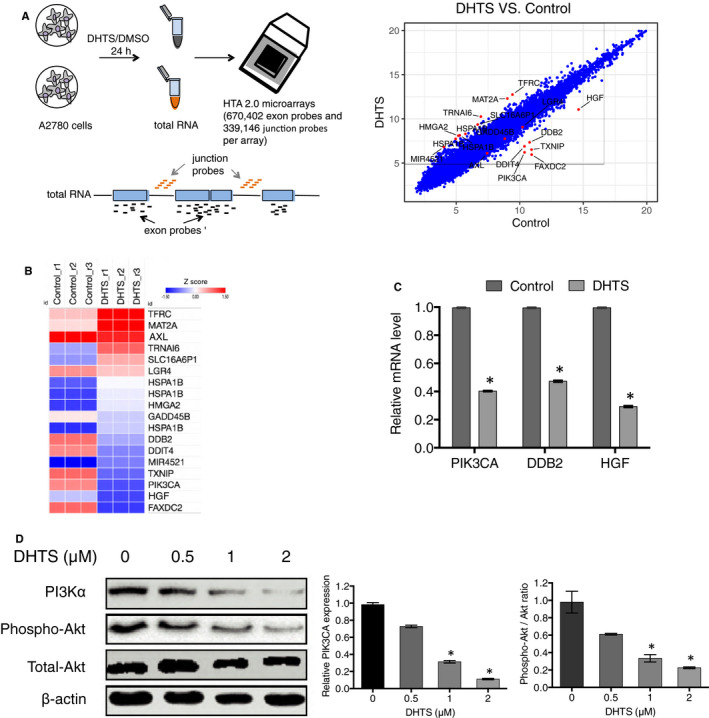
Critical role of PI3K/AKT signalling pathway in DHTS induced A2780 ovarian cancer cell death. A, Scatter plot comparing global gene expression profiles in DHTS‐treated and DMSO‐treated A2780 ovarian cancer cells. Each dot presented on the graph corresponds to one transcript. A2780 cells were treated with DHTS (2 μM) or DMSO (Control) for 24 h, followed by total RNA isolation and Affymetrix HTA 2.0 array. B, Heat map of differentially expressed genes found in DHTS‐treated vs. DMSO‐treated A2780 cells. C, mRNA expression levels of PIK3CA, DDB2 and HGF detected by real‐time PCR. A2780 cells were treated with DHTS (2 μM) or DMSO for 24 h, followed by mRNA isolation and real‐time PCR. D, Regulation of PI3K/AKT signalling pathway in A2780 ovarian cancer cells treated with DHTS. Western blotting was performed to determine the expression or phosphorylation of PI3Kα, p‐AKT and AKT in A2780 cells treated with DHTS (0, 0.5, 1 and 2 μM) for 48 h. *P < .05 vs. untreated control

To characterize the suppression effect mediated by DHTS on ovarian cancer cell proliferation, migration and invasion, we performed gene expression profiling of DHTS‐treated (2 μM for 24 hours) and control DMSO‐treated A2780 cells cultured in vitro by Affymetrix Human Transcriptome Array 2.0 (HTA 2.0, Illumina). We hybridized total RNA from cells to HTA 2.0 genechips, each chip contains both exon (~670 k) and exon‐exon (~340 k) junction probes. A total of 509 differentially expressed genes, 385 genes up‐regulated and 124 genes down‐regulated, were identified to be associated with DHTS treatment compared to the control cells (Figure 3A). Critically, of these events, six protein‐coding genes, phosphatidylinositol‐4, 5‐bisphosphate 3‐kinase catalytic subunit alpha (PIK3CA), damage‐specific DNA‐binding protein 2 (DDB2), hepatocyte growth factor (HGF), thioredoxin‐interacting protein (TXNIP), fatty acid hydroxylase domain containing 2 (FAXDC2) and DNA damage inducible transcript 4 (DDIT4) were identified as most dramatically affected genes (Figure 3B). Among these targeting genes, we found that PIK3CA, DDB2 and HGF mRNA expression were significantly down‐regulated in A2780 cells exposed to DHTS (Figure 3C). In addition, Western blotting analysis showed that PI3Kα expression and its downstream protein AKT phosphorylation in A2780 treated with DHTS were also significantly down‐regulated (Figure 3D). These data implied a potential role of DHTS in the regulation of PIK3CA gene transcription in ovarian cancer cells.

### 3.4 DHTS suppressed proliferation of ovarian cancer cells by transcriptionally regulating endogenous PIK3CA gene expression

**FIGURE 4:**
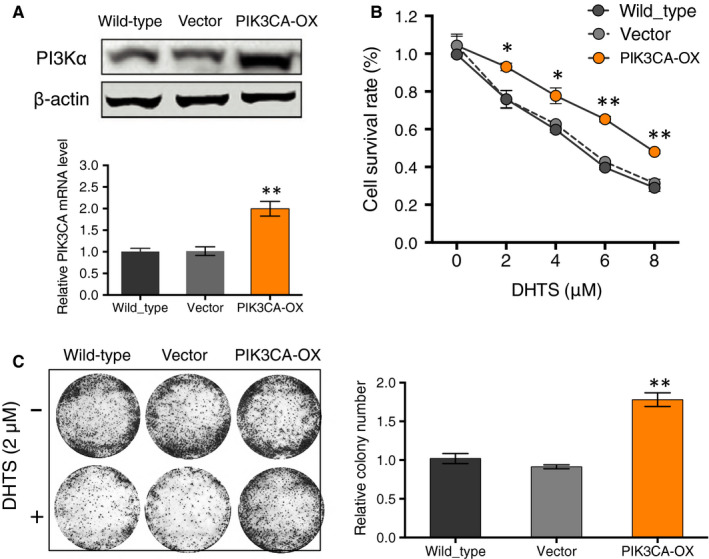
Overexpression of PIK3CA increases the viability and colony formation of DHTS‐treated A2780 ovarian cancer cells. A, Ectopic expression of PIK3CA in A2780 cells. PIK3CA expression level of A2780 cells that was transfected with an empty vector (Vector) or with a vector encoding PIK3CA (PIK3CA‐OX) was detected by real‐time PCR and Western blotting. B, Effect of PIK3CA overexpression on proliferation in DHTS‐treated A2780 cells after indicated plasmid transfection by MTT assay. Cells were treated with various concentrations of DHTS (0, 2, 4, 6 and 8 μM) for 24 h. C, Colony formation of A2780 cells stably expressing indicated plasmids. Cancer cells were treated with DHTS (2 μM) for 48 h and in vitro cultured for another 2 weeks for colony forming. The surviving colonies were stained with crystal violet and counted with ImageJ (colony counter). Data represent means ± SEM of triplicate samples. *P < .05 and **P < .01 vs. untreated control or wild‐type

Preclinical investigations have suggested that the PI3K/AKT pathway is frequently activated in ovarian cancer.^32^, ^33^, ^34^ Thus, this pathway is regarded as an attractive candidate for cancer therapeutic interventions. The inhibitors targeting different components of this pathway are at different stages of clinical development. From the above findings, we asked whether DHTS‐inhibited proliferation of A2780 cancer cells was via down‐regulating PIK3CA gene transcription. To address this, ovarian cancer A2780 cells were transfected with an overexpression plasmid (PIK3CA‐OX) encoding wild‐type PIK3CA gene or an empty plasmid as negative control (vector). After transfection, we performed qPCR and Western blotting to determine PIK3CA expression and we observed exogenous wild‐type PIK3CA significantly increasing in PIK3CA‐OX cells (Figure 4A). Meanwhile, cell proliferation assay showed that A2780 cells stably expressing exogenous PIK3CA developed resistance to DHTS induced anti‐proliferation (Figure 4B). Additionally, clone formation assay showed that the clone‐forming ability of stable exogenous PIK3CA expressing‐A2780 cells was significantly elevated as compared to control groups in the presence of 2 μM DHTS (Figure 4C). These data supported that DHTS treatment significantly down‐regulated the PIK3CA transcription and protein levels, and followed activity of PI3K/AKT signalling pathway, leading to anti‐proliferation of ovarian cancer cells.

### 3.5 Overexpression of PIK3CA gene in A2780 ovarian cancer cells confers resistance to DHTS‐prohibited cell migration and invasion

**FIGURE 5:**
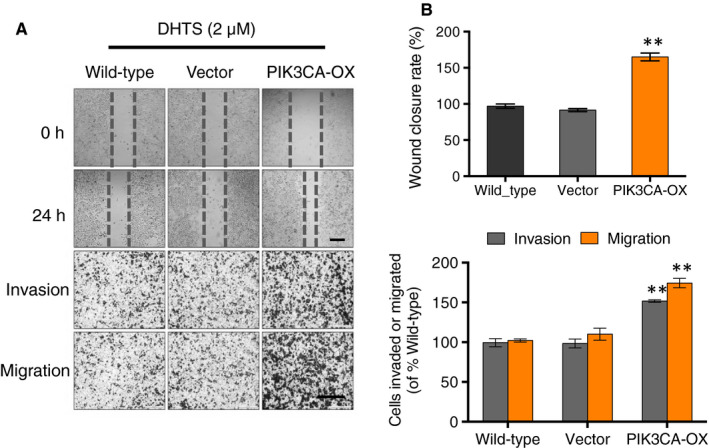
Overexpression of PIK3CA gene increases cell migration and invasion of DHTS‐treated A2780 ovarian cancer cells. (A, B) Effect of PIK3CA overexpression on migration and invasion in DHTS‐treated A2780 cells after indicated plasmid transfection by wound‐healing assay (top) and transwell chamber migration and invasion assays (bottom). Cells were treated with DHTS (2 μM) for 24 h. Bar scale = 200 μm, **P < .01 vs. untreated control

Next, we investigated whether DHTS inhibits ovarian cancer cell migration and invasion by regulating PIK3CA transcription. Here, we used wound healing and transwell chamber assays on wild‐type, empty vector transfected (vector) and PIK3CA overexpressing (PIK3CA‐OX) A2780 cells. In the scratch wound healing assay, the wound closure rate of empty vector transfected A2780 cells was similar to that of the wild‐type cells, whereas PIK3CA‐overexpressing A2780 cells showed a significantly increased motility to two control cell lines in response to a 24 hours DHTS treatment (Figure 5A,B upper panel). In transwell assay, the cells were plated in the upper chamber of the filters and were allowed to migrate for 24 hours under DHTS. The number of wild‐type cancer cells that had migrated to the underside of the filters was similar to that of the empty vector transfected cells. However, PIK3CA‐overexpressing A2780 cells showed significantly increased migration and invasion by 30%‐50% compared to other two different subcell lines (Figure 5A,B lower panel). These data indicated that PIK3CA is required in the process of migration and invasion of ovarian cancer cells treated by DHTS. On the other hand, DHTS could inactivate PI3K pathway to reduce the motility of ovarian cancer cells.

### 3.6 DHTS sensitizes ovarian cancer cells to platinum therapy

**FIGURE 6:**
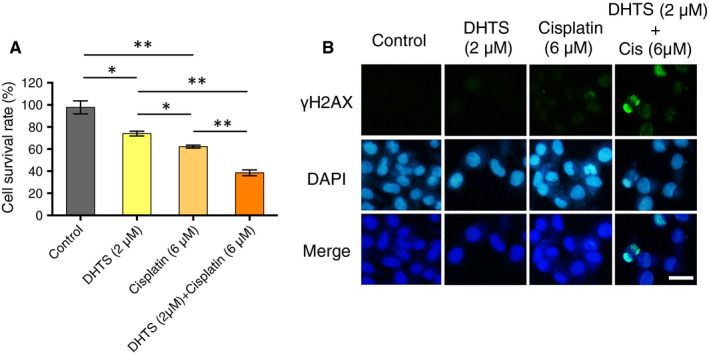
DHTS sensitizes A2780 ovarian cancer cells to cisplatin by inducing DNA damage. (A) Anti‐proliferative effect of different compounds in A2780 cells by MTT assay. Cell lines were treated with DMSO (control), DHTS (2 μM), cisplatin (6 μM) or a combination of DHTS and cisplatin for 48 h. (B) Levels of cellular γH2AX foci in A2780 cancer cells by immunofluorescence. A2780 cells were treated with different agents for 24 h, followed by γ‐H2AX antibody staining. Bar scale = 50 μm, *P < .05 and **P < .01 vs. untreated control

The standard therapy of clinical management of advanced ovarian cancer following debulking surgery is chemotherapy with a platinum‐based agent (cisplatin or carboplatin) and a taxane‐based agent (paclitaxel or docetaxel).^35^, ^36^ In addition, previous studies have shown that inhibition of PI3K signalling pathway sensitized ovarian cancer cell lines to the anti‐tumour effects of platinum compounds. We reasoned that the anti‐tumour effects of cisplatin could be further enhanced in combination with DHTS, because of PIK3CA down‐regulating activity of DHTS. Indeed, we found that DHTS in combination with cisplatin exhibited enhanced cytocidal effect on A2780 cells (Figure 6A). Then, we investigated whether DNA damage was involved in the chemosensitization effect of DHTS. A2780 cells were treated with either DHTS (2 μM), cisplatin (6 μM), or both for 24 hours, followed by immunofluorescence staining. Interestingly, DNA double‐strand break marker, formation of γH2AX foci was highly induced in combination‐treated cells as compared to single agent or DMSO‐treated cells (Figure 6B). Our data suggested that DHTS significantly increased the sensitivity of ovarian cancer cells to cisplatin through the induction of DNA damage.

### 3.7 DHTS inhibits ovarian cancer cell proliferation and metastasis in zebrafish ovarian cancer model

**FIGURE 7:**
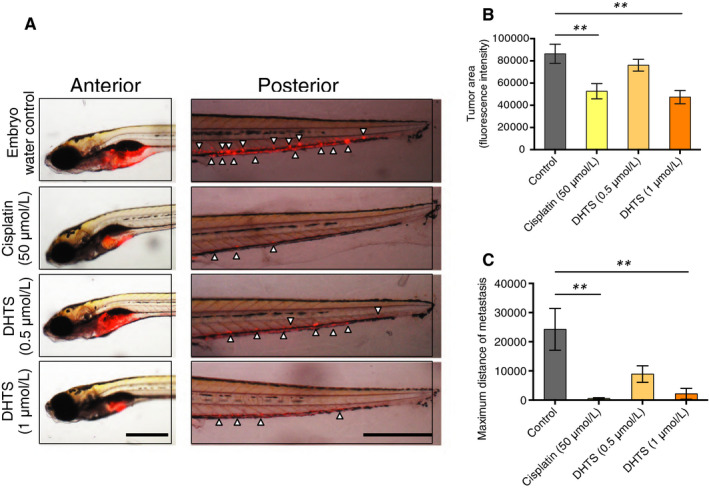
Inhibition of tumour growth and metastasis by DHTS in zebrafish cancer model. A, Effect of DHTS and cisplatin on tumour growth and metastasis in zebrafish xenografts. Cancer‐bearing zebrafish embryos were treated with DHTS (0.5 and 1 μmol/L) or cisplatin (50 μmol/L). Arrowheads indicate metastatic tumour cells in the distal parts of the fish body (Bar scale = 500 μm). B, Quantification of tumour volume (n = 10/group). c Averages of maximal distances of metastatic foci (n = 10/group). Data represent means ± SEM. **P < .01 vs. untreated control

To assess the potentiating effect of DHTS on anti‐tumour activity in vivo, a zebrafish xenotransplantation model was used. A2780 ovarian cancer‐bearing zebrafish embryos were treated with different doses of DHTS (0.5 μmol/L or 1 μmol/L), cisplatin (50 μmol/L) as positive control, or zebrafish embryo water as negative control. As we expected, DHTS effectively blocked tumour cell proliferation and significantly suppressed primary tumour growth (Figure 7A left panel and B). A higher dose of DHTS (1 μmol/L) exhibited a better anti‐tumour efficacy compared with control group (Figure 7B). Consistent with the inhibition of tumour cell proliferation, DHTS also sufficiently blocked tumour metastasis (Figure 7A right panel and C). These data demonstrated that DHTS can significantly in vivo inhibited primary ovarian cancer growth and followed tumour metastasis.

## 4 DISCUSSION

Ovarian cancer is the leading cause of death from all gynecologic cancers.^1^ Currently, treatment for ovarian cancer involves platinum‐based chemotherapy that has many limitations, such as rapid clearance, severe side effects, unfavourable biodistribution and drug resistance.^37^, ^38^ There is an urgent unmet clinical need in developing new therapies for patients with ovarian cancer. In the past decade, the advent of immunotherapeutic and molecularly targeted agents has dramatically revolutionized the therapeutic landscape for cancer treatment. For example, PARP inhibitor olaparib showed a 70% reduction in the risk of progression or death for ovarian cancer patients with BRCA1/2 mutation.^39^ Rucaparib, a novel inhibitor of PARP, demonstrated a 56% response rate in patients with high‐grade, recurrent, platinum‐sensitive ovarian carcinoma and is evaluated in a phase 3 trial.^40^ Our previous study has shown the promise of combining inhibitors of PI3K and PARP as treatment for ovarian cancer.^41^ In addition, several checkpoint blocking antibodies, such as those directed against CTLA‐4 and PD‐1, have been developed and are being tested clinically in patients with ovarian cancer.^42^, ^43^

Currently, accumulating evidence suggests that phyto‐active compounds hold promise as adjuvants of traditional chemotherapy and may be helpful for the chemo‐prevention of ovarian cancer.^44^, ^45^ In our study, we found that suppression of PI3K pathway activity by DHTS was accompanied by increased abundance of the DNA damage marker γH2AX in well‐established cell models of constitutively active PI3K ovarian cancer. Previous works have reported that gain of function (GOF) mutations of PIK3CA or loss of PTEN led not only to PI3K signalling pathway activation, but also accumulation of DNA DSBs, as well as PTEN‐deficient cells being susceptible to killing by a combination of PI3K inhibitors and genotoxic stress.^46^, ^47^, ^48^, ^49^ Interestingly, the PTEN‐deficient ovarian cancer cell line A2780 examined in the present study exhibited enhanced sensitivity by combined DHTS and cisplatin treatment. In addition, high dose DHTS exhibited less toxicity in normal ovarian cells, suggesting the effect was specific to ovarian cancer cells harbouring mutations that up‐regulate PI3K pathway activity.

Abnormal activation of PI3K pathway has been shown to promote migration and invasion through different mechanisms, such as regulation of VEGF and MMP‐9.^50^, ^51^ Here, we observed that the suppressed migration and invasion ability of A2780 and OV2008 was induced by down‐regulating PI3K activation. Meanwhile, overexpression of wide‐type PIK3CA gene could restore the migration and invasion abilities of DHTS‐treated cells. All these findings indicate that DHTS regulates PIK3CA gene at the transcriptional level, rather than directly targeting p110α protein. Interestingly, a previous study demonstrated that cryptotanshinone, similar to DHTS in structure, was found to inhibit phosphorylation of AKT and S6RP in human rhabdomyosarcoma and prostate cancer cells by not altering the protein expression of p110.^52^ Undoubtedly, it is of great importance to elucidate how DHTS regulates PIK3CA transcription, as this may provide direction for the development of more promising DHTS analogues for ovarian cancer treatment.

## 5 CONCLUSIONS

In this study, our study has demonstrated that targeting PI3K/AKT signalling pathway activity using DHTS in ovarian cancer represents a promising therapeutic strategy. We suggest the administration of DHTS, as an adjuvant for chemotherapeutic agents, for ovarian cancer patients with high PI3K activation to overcome ovarian cancer invasion and metastasis.

## CONFLICT OF INTEREST

The authors confirm that there are no conflicts of interest.

## AUTHOR CONTRIBUTIONS

Lin Zhang and Weiling Li involved in conceptualization; Xiaoqing Wang, Guoqiang Jiang, and Xiao Xu involved in data curation; Xiaoqing Wang and Xiao Xu involved in formal analysis; Lin Zhang and Weiling Li involved in funding acquisition; Weiling Li involved in investigation; Xiaoqing Wang and Guoqiang Jiang involved in methodology; Weiling Li involved in project administration; Lin Zhang and Weiling Li involved in resources; Xiao Xu involved in software; Weiling Li involved in supervision; Xiaoqing Wang, Cuili Zhang, and Likun Liu involved in validation; Xiao Xu involved in visualization; Xiaoqing Wang involved in writing—original draft; Jian Kang, Jing Wang, Lawrence Owusu, Liye Zhou, and Weiling Li involved in writing—review and editing. All authors reviewed the manuscript.

## Data Availability

The data that support the findings of this study are available from the corresponding author upon reasonable request.
